# Intrusive imagery in severe health anxiety: Prevalence, nature and links with memories and maintenance cycles

**DOI:** 10.1016/j.brat.2010.05.008

**Published:** 2010-08

**Authors:** Kate Muse, Freda McManus, Ann Hackmann, Matthew Williams, Mark Williams

**Affiliations:** a University of Oxford, Department of Psychiatry, Warneford Hospital, Oxford OX3 7JX, United Kingdom; b Oxford Cognitive Therapy Centre, Warneford Hospital, Oxford OX3 7JX, United Kingdom

**Keywords:** Intrusive imagery, Images, Health anxiety, Hypochondriasis

## Abstract

Increased understanding of the nature and role of intrusive imagery has contributed to the development of effective treatment protocols for some anxiety disorders. However, intrusive imagery in severe health anxiety (hypochondriasis) has been comparatively neglected. Hence, the current study investigates the prevalence, nature and content of intrusive imagery in 55 patients who met DSM-IV-TR (APA, 2000) criteria for the diagnosis of hypochondriasis. A semi-structured interview was used to assess the prevalence, nature and possible role of intrusive imagery in this disorder. Over 78% of participants reported experiencing recurrent, distressing intrusive images, the majority (72%) of which either were a memory of an earlier event or were strongly associated with a memory. The images tended to be future orientated, and were reliably categorised into four themes: i) being told ‘the bad news’ that you have a serious/life threatening-illness (6.9%), ii) suffering from a serious or life-threatening illness (34.5%), iii) death and dying due to illness (22.4%) and iv) impact of own death or serious illness on loved ones (36.2%). Participants reported responding to experiencing intrusive images by engaging in avoidance, checking, reassurance seeking, distraction and rumination. Potential treatment implications and links to maintenance cycles are considered.

Severe and persistent health anxiety is diagnosed as the somatoform disorder ‘hypochondriasis’ in DSM-IV-TR (APA, 2000). It has however been argued that the underlying cognitive processes are more consistent with those in anxiety disorders (Mayou, Kirmayer, Simon, Kroenke, & Sharpe, 2005; Noyes, 1999; Olatunji, Deacon, & Abramowitz, 2009). Furthermore, as a label, the diagnosis of ‘hypochondriasis’ has negative connotations. Thus it may be less pejorative and more clinically useful to conceptualise hypochondriasis as severe and persistent health anxiety, lying at the far end of a continuum that has mild health anxiety at its other end. Hence, in the current paper the term ‘health anxiety’ is used instead of ‘hypochondriasis’.

Severe and persistent health anxiety is characterised by pre-occupation with the fear of having a serious disease, which persists in spite of appropriate medical reassurance. Epidemiological studies report that up to nine per cent of patients in general medical practice clinics experience health anxiety (Creed & Barsky, 2004; Gureje, Ustun, & Simon, 1997) and the prevalence in the general population has been reported to be as high as five per cent (Asmundson, Taylor, Sevgur, & Cox, 2001). Health anxiety not only causes great suffering for the patient and those around them but is also costly in terms of higher medical care utilisation (Barsky, Ettner, Horsky, & Bates, 2001). Hence, it remains a priority to understand the aetiology and maintenance of health anxiety and to develop effective treatments. Although imagery research has contributed both to the understanding and treatment of a number of anxiety disorders (Hirsch & Holmes, 2007), there has been little previous investigation of imagery in health anxiety. Hence the current paper explores the prevalence and nature of intrusive imagery in health anxiety for the purpose of providing further insight into the aetiology, maintenance and treatment of the disorder.

Intrusive imagery is a hallmark feature of PTSD (Brewin & Holmes, 2003; Ehlers, Hackmann, & Michael, 2004; Steil & Ehlers, 2000) and has been reported to affect the majority of patients with social phobia (Hackmann, Clark, & McManus, 2000), agoraphobia (Day, Holmes, & Hackmann, 2004), simple phobia (Pratt, Cooper, & Hackmann, 2004) and OCD (Speckens, Hackmann, Ehlers, & Cuthbert, 2007). Previous research suggests that such intrusive images are often linked to memories of adverse events that occurred in childhood or around the time of onset of the disorder (Day et al., 2004; Hackmann et al., 2000; Speckens et al., 2007), thus providing insight into the development of the disorder and leading the development of effective imagery re-scripting treatment techniques (Holmes, Arntz, & Smucker, 2007; Hunt & Fenton, 2007; Wild, Hackmann, & Clark, 2007, 2008).

Intrusive imagery has also been demonstrated to play a role in the maintenance of some anxiety disorders. For example, Hirsch, Clark, Mathews, and Williams (2003) reported that holding in mind a negative rather than neutral self image increased socially phobic participants’ anxiety and had a detrimental effect on their self-impression and observer rated performance. Maladaptive responses to intrusive images have also been shown to contribute to the maintenance of anxiety disorders (Hackmann & Holmes, 2004). For example, Steil and Ehlers (2000) found that rumination, thought suppression and distraction in response to intrusive images was associated with PTSD severity and Speckens et al. (2007) reported that patients with OCD responded to intrusive images with obsessing, neutralising, distraction, avoidance and reassurance seeking. The prevalence and nature of imagery in patients with health anxiety, however, is not yet known. Nor is it clear whether they respond to intrusive imagery with strategies that have been hypothesised to maintain anxiety disorders, such as checking, reassurance seeking, avoidance, distraction or rumination (Abramowitz & Moore, 2007; Salkovskis & Warwick, 2001; Taylor & Asmundson, 2004).

To date there has only been one previous study of intrusive imagery in patients with health anxiety (Wells & Hackmann, 1993). This preliminary study reported that such patients had images which centred on themes of the self, death and illness, and were often linked to memories of adverse events. However, this study only included ten patients who were selected specifically because they reported experiencing imagery, rather than being a representative sample. Hence, it cannot give any indication of the prevalence of intrusive of imagery in health anxiety. The current study aims to build upon these initial findings by using a semi-structured interview to examine the occurrence and nature of intrusive imagery in a larger sample of patients who meet DSM-IV-TR (APA, 2000) criteria for the diagnosis of hypochondriasis.

The current study had three main aims:

1. To determine the prevalence and nature of recurrent intrusive imagery in participants with health anxiety, and to examine the content and characteristics of the imagery in health anxiety (frequency, recurrence, time code, vividness, perspective and associated distress).
2. To determine whether intrusive images are associated with specific memories, and if so whether the events that the memories relate to cluster in time around the onset of participants’ health anxiety. If images are associated with memories, to establish the level of distortion of the image in relation to the memory of the actual event (i.e., the degree to which the image is an accurate representation of the memory of the event).
3. To determine whether participants respond to intrusive images by engaging in behavioural responses hypothesised to maintain health anxiety (e.g., avoidance, reassurance seeking etc.).

## Method

### Participants and recruitment

Participants were invited to take part in the study after attending an assessment to participate in a randomised controlled trial of mindfulness-based cognitive therapy (MBCT) for health anxiety (McManus, Williams, Surawy, & Muse, in preparation). Diagnoses were established for the purposes of the randomised controlled trial, by a trained assessor, using the Structured Clinical Interview for Diagnosis (First, Spitzer, Gibbon, & Williams, 1997). Participants were given information about the study and signed a written consent form.

### Measures

#### Whiteley Index

The Likert-scale version of the Whiteley Index (Pilowsky, 1967; Welch, Carleton, & Asmundson, 2009) is a 14-item self-report questionnaire measuring health anxiety, and has been demonstrated to have good validity and reliability (Welch et al., 2009).

#### Short Health Anxiety Inventory

The Short Health Anxiety Inventory (Salkovskis, Rimes, Warwick, & Clark, 2002) is an 18-item self-report questionnaire measuring health anxiety which has been shown to be reliable, to have a high internal consistency and to have good sensitivity/specificity (Salkovskis et al., 2002).

#### Beck Depression Inventory

The Beck Depression Inventory-II (Beck, Brown, & Steer, 1996) is a widely used 21-item self-report measure of depression that has been demonstrated to have good reliability and validity (Beck, Steer, & Carbin, 1988).

#### Beck Anxiety Inventory

The Beck Anxiety Inventory (Beck & Steer, 1990) is a widely used 21-item self-report measure of anxiety which has a high internal consistency, test–retest reliability and convergent validity (Beck, Epstein, Brown, & Steer, 1988).

#### Semi-structured interview

The semi-structured interview was based on those used in previous studies (e.g., Day et al., 2004; Hackmann et al., 2000; Speckens et al., 2007) and covered the following areas:

##### Interview section 1: prevalence, nature and content

Participants were asked to focus on their experiences of being anxious about their health in order to identify related intrusive imagery. Imagery was defined as a multi-sensory experience which could include any of the five modalities (visual, sounds, bodily sensations, taste and smell). Those who experienced intrusive imagery were asked to identify their most significant/distressing image and to evoke this image and describe it. Participants were then asked to respond to all subsequent questions in relation to this ‘index image’ only.

Participants’ descriptions of the index image were transcribed verbatim and analysed using a content analysis approach (Stemler, 2001). Participants’ images were initially independently reviewed by two raters (first and fourth authors) in order to identify recurrent features. These features were then compared and any differences reconciled through discussion to produce a final summary list of themes. Finally, the two raters independently coded each of the images as fitting into one or more of the defined themes. Reliability of this coding was established by comparing agreement between raters.

Whether the image was recurrent rather than a one-off was established, and the frequency with which the image had occurred in the last week was noted. Images were then categorised as relating to the past, present or future and participants rated how vivid and distressing the image was from 0 ‘not at all’ to 100 ‘extremely’. Participants also rated the predominant viewpoint in the image using the following scale: from ‘−3 field perspective’, defined as “looking out through your own eyes, observing details of what is going on around you”, through ‘0 interchangeable perspective’ defined as “alternating between the two perspectives”, to ‘+3 observer perspective’, defined as ‘looking at yourself from the outside/an external point of view’.

##### Interview section 2: links to memories

Participants focused on their index image and were asked whether the image was a memory of an event. If participants reported that the image was a memory of an event they were asked to describe the event and rate how distorted the image was in relation to their memory of the actual event on a scale from 0 (not at all distorted: the image is an accurate representation of the event) to 100 (very distorted: the depiction of the event is inaccurate in the image e.g., important elements are different). If participants reported that the image was not a memory of an event, they were asked if it was associated with any memories in terms of the sensations, emotions or thoughts evoked by the image. If the image was associated with a memory, participants rated how strongly the image and memory were linked on a 0–100 scale. The chronology of the event that the memory related to and the onset of the participant’s health anxiety were identified, and if the event occurred after the onset of health anxiety, participants rated whether the identified event had an impact on the severity of their symptoms at that time (from −3 ‘negative impact’ to +3 ‘positive impact’).

##### Interview section 3: responses to images

Participants were presented with the statement: “usually when I experience the distressing image…” followed by five behavioural responses hypothesised to maintain health anxiety (Salkovskis & Warwick, 2001; Taylor & Asmundson, 2004). These were: rumination “I keep thinking about the image after it is gone”; avoidance “In my mind I try and push it away”; distraction “I find it so unpleasant I have to distract myself and not notice it”; checking “I check my body/health/symptoms” and reassurance seeking “I seek reassurance about my health”. The extent to which participants agreed with the statement was measured on a 0 (strongly disagree)–6 (strongly agree) scale.

### Procedure

Interviews were audio recorded and lasted approximately 30 min. Where ratings on visual analogue scales were required, participants were shown the relevant scale with anchors.

## Results

### Demographics/study population

Fifty-five participants took part in the study. The demographics for this sample are outlined in Table 1. All participants met DSM-IV-TR (APA, 2000) criteria for the diagnosis of hypochondriasis. From Table 1 it can be seen that the levels of health anxiety, depression and anxiety are similar to those reported in other samples of patients with health anxiety (Barsky & Ahern, 2004; Barsky, Wyshak, & Klerman, 1990; Salkovskis et al., 2002). There were no significant differences in the demographic characteristics or symptom severity between participants who experienced mental images and those who did not (see Table 1).

### Prevalence and nature of imagery

Forty-three participants (78.2%) reported experiencing intrusive imagery when feeling anxious about their health. Only the results from these 43 participants are discussed further. All participants reported that their index image was recurrent, in that the same image was experienced repeatedly. The mean number of times the index image was experienced during the previous week was 3.77 (SD = 6.41). The mean vividness and distress ratings of the index image were 57.69 (SD = 27.43) and 65.71 (SD = 30.01) respectively. Fifteen participants (34.88%) experienced their image from a ‘field’ perspective (looking out through your own eyes), twenty (46.51%) experienced their image from an observer perspective (looking at yourself from the outside) and eight reporting experiencing their image from both perspectives interchangeably (18.60%). Thirty-seven (86.05%) participants categorised their image as relating to the future, three (6.98%) as relating to the past and three (6.98%) as relating to the present.

### Content of imagery

Content analysis revealed that the 42 intrusive images could be categorised into four themes: i) being told ‘the bad news’ that you have a serious/life-threatening illness (6.9%), ii) suffering from a serious or life-threatening illness (34.5%), iii) death and dying due to illness (22.4%) and iv) impact of own death or serious illness on loved ones (36.2%). See Table 2 for examples and quotes.

It was possible for an image to be classified into more than one theme, meaning that the 42 images were classified into themes a total of 58 times, with 26 images being classified into one theme only, and 16 being classified into two themes. All of the 16 images classified into two themes fell under ‘impact on loved ones’ as well as another theme. One miscellaneous image could not be categorised into any theme. The percentage coding agreement among raters was 95%, with 3 initial discrepancies occurring. Discrepancies were discussed to reach consensus.

### Links to memories

Of the forty-three participants who experienced intrusive images, 31 (72.09%) reported that their index image either was a memory or was associated with a memory. Of these 31, 11 (35.48%) reported that their index image was a memory of an actual event such as a prior experience of illness. The mean rating (from 0 ‘no distortion’ to 100 ‘very distorted’ scale) for how distorted the image was in relation to the memory of the actual event was 32.73 (SD = 36.35), indicating that the memory of the event was not highly distorted in the image. A typical example of the way in which the image was a distortion of the memory/actual event was for the event to be of a significant other’s illness, but for the image to be of seeing one’s self in place of the ill person. For example seeing yourself in hospital dying of cancer in surroundings that resemble those in which you watched your father dying of cancer – such an image is a memory of the father’s death but is not an accurate recalling of it and thus can be said to be significantly distorted. For the remaining twenty participants (64.51%), the image was associated with a memory of an earlier event in terms of the sensations, emotions and thoughts experienced, but was not a memory of it. One participant, for example, imagined herself ill in hospital with the surrounding people cross with her for being ill. She associated this image with feelings of blame during domestic abuse and concerns that had been raised then about possible longer-term damage from resultant injuries. The mean rating for how strongly the images and memories were associated was 68.38 (SD = 29.07) (on the scale from 0 = ‘no association’ to 100 ‘very strongly associated’), indicating that the images were strongly linked with the associated memory.

**Fig. 1:**
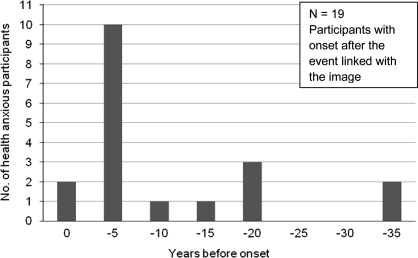
The temporal relationship between participants’ onset of health anxiety and the events associated with their intrusive images.

Of the 31 participants whose image was a memory of an event, or was associated with a memory, 19 reported that their health anxiety symptoms started after the event associated with the image. The majority of these participants (*n* = 12, 63.16%) reported that the event depicted in the memory occurred within 5 years prior to the onset of health anxiety, the other seven participants (36.84%) reported that the event occurred between 7 and 33 years before the onset of their health anxiety, usually during childhood (see Fig. 1).

For the twelve participants who already had health anxiety symptoms at the time of the event associated with the image, eight reported that their symptoms worsened after the event and four did not notice an impact of the event on the severity of their health anxiety symptoms.

### Responses to imagery

Participants’ rated how much they usually engaged in possible health anxiety maintaining behaviours in response to experiencing intrusive imagery (on a 0–6 scale) as follows: reassurance seeking (*M* = 4.40, SD = 1.86), checking their body/health/symptoms (*M* = 4.36, SD = 1.87), avoidance (*M* = 4.10, SD = 1.63), distraction (*M* = 3.38, SD = 2.06) and rumination (*M* = 3.31, SD = 1.83).

## Discussion

### Prevalence, nature, content and responses to images

Over 78% of participants with health anxiety reported experiencing intrusive imagery, which compares to reported rates of 100% in social phobia (Hackmann et al., 2000), 100% in agoraphobia (Day et al., 2004), 81% in OCD (Speckens et al., 2007) and 69% in specific phobia (Pratt et al., 2004). Participants’ experienced their index image an average of 3.77 times per/week, which is lower than the reported five times per/week in PTSD (Hackmann, Ehlers, Speckens, & Clark, 2004) and ten times per/week in OCD (Speckens et al., 2007). This disparity could reflect the fact that intrusive thoughts/images are a central diagnostic feature of PTSD and OCD but not of other anxiety disorders. However, because few imagery studies have reported frequency of occurrence, it is unknown whether intrusive imagery is less frequent in health anxiety than in all other anxiety disorders or only than OCD and PTSD. The mean distress associated with participants’ index images was 65.71, which is comparable to reported distress ratings of 60 in specific phobia (Pratt et al., 2004), 70 in PTSD (Hackmann et al., 2004) and 80.9 in OCD (Speckens et al., 2007).

Previous research has reported an observer perspective bias in intrusive imagery in social phobia (Hackmann, Surawy, & Clark, 1998), body dysmorphic disorder (Osman, Cooper, Hackmann, & Veale, 2004) and eating disorders (Somerville, Cooper, & Hackmann, 2007). However, it has been suggested that this bias may only be evident in disorders where social evaluative or appearance related concerns are primary (Wells & Papageorgiou, 1999). Consistent with this suggestion, and with findings from other studies of imagery in disorders where social evaluation is not a core feature e.g., simple phobia and OCD (Pratt et al., 2004; Speckens et al., 2007), the current study did not find an observer perspective bias in the images of health anxious participants, with their images being from both observer and field perspectives.

Participants’ images centred on themes of death and serious illness. Given that the overestimation of the probability and cost of illness and death are key cognitive components of health anxiety (Barsky et al., 2001; Salkovskis & Clark, 1993), it is perhaps not surprising that intrusive images also centre on this theme. However, it is worth noting that the majority of participants (86.05%) classified their image as relating to the future. Imagining a future event has been shown to increase an individual’s perception of the probability that the imagined event will occur (Carroll, 1978; Sherman, Cialdini, Schwartzman, & Reynolds, 1985). It is therefore possible that the recurrent, future-oriented intrusive images along themes of illness and death serve to maintain anxiety about health by increasing participants’ estimation of the likelihood of these events occurring. Further research could assess the impact of intrusive imagery on health anxious patients’ estimations of the probability and cost of future illnesses.

Participants’ responses suggested that they tended to respond to intrusive images by engaging in avoidance, checking, reassurance seeking, distraction and rumination. Responding to images with maladaptive cognitive/behavioural avoidance strategies has been implicated in the maintenance of intrusive phenomena in anxiety disorders (Lavy & Van Den Hout, 1994; Lawrence, Fauerbach, & Munster, 1996; Salkovskis & Campbell, 1994). Furthermore, avoidant responses to intrusive imagery in OCD and PTSD have been shown to predict the persistence of the disorder (Ehlers & Steil, 1995; Ladouceur et al., 2000; Steil & Ehlers, 2000) and such maladaptive strategies have been suggested as maintaining health anxiety (Abramowitz & Moore, 2007; Salkovskis & Warwick, 2001; Taylor & Asmundson, 2004). Frequently engaging in maladaptive behaviour in response to intrusive imagery could therefore maintain both the intrusive phenomena and anxiety by prompting maladaptive cycles of responding. Whilst the present study did not directly measure the actual impact that intrusive imagery had on subsequent behaviour (e.g., whether it increased frequency of reassurance seeking), the initial results presented here suggest that this is an area which future research could usefully delineate.

### Links to memories

The majority of participants’ intrusive images (72%) were reported to be either a memory of an earlier event or strongly associated with a memory. This is consistent with previous research reporting that an associated memory was identified by 100% of participants with agoraphobia (Day et al., 2004), 96% with social phobia (Hackmann et al., 2000), 79% with OCD (Speckens et al., 2007) and 55% with specific phobia (Pratt et al., 2004). The majority of participants (61.29%) who identified a link between their image and a memory reported that the associated event occurred prior to the onset of their health anxiety, either in childhood or within the 5 years prior to the onset of health anxiety. The majority of participants whose health anxiety began before the event associated with the image reported that their symptoms worsened after this event. These findings are in line with other studies reporting that memory events associated with intrusive images often date to childhood (Day et al., 2004; Osman et al., 2004; Pratt et al., 2004; Somerville et al., 2007) and that associated memories often cluster around the onset of the disorder, although this clustering has been reported more consistently in social phobia than in other anxiety disorders (Day et al., 2004; Hackmann et al., 2000; Speckens et al., 2007). Given the small number of participants for whom associations between memories linked to their images and the onset or exacerbation of health anxiety was relevant, firm conclusions cannot be drawn. However, the preliminary data reported here suggests that enquiring about memories of adverse events linked to images may be clinically relevant and could provide insight into the development and/or exacerbation of health anxiety for some patients.

### Clinical implications

Increased understanding of the role of imagery in other anxiety disorders has contributed to the development of effective treatment techniques, which have been demonstrated to have therapeutic benefits as stand-alone interventions and when incorporated into multi-component CBT protocols (e.g., Arntz, Tiesema, & Kindt, 2007; Clark et al., 2006; Grunert, Weis, Smucker, & Christianson, 2007; Holmes, Arntz, et al., 2007; Hunt & Fenton, 2007; Wild et al., 2007, 2008). Given that research into psychological treatments for health anxiety is at an early stage and the superiority of any one approach has not yet been demonstrated (Thomson & Page, 2007), it is a particular priority to focus on improving outcomes from psychological treatments for health anxiety. The current study’s findings indicate a number of areas which may have relevance for refining treatments for the health anxiety.

Participants’ images centred on themes of future illness and death, which is consistent with the inflated perception of the probability and cost of illness observed in health anxiety. Predictions about likelihood and cost of becoming ill and/or dying are difficult to disconfirm, which will present challenges for direct imagery interventions which seek to disconfirm the content or meaning of images. Hence, future research could usefully investigate effects of indirect imagery interventions on intrusive imagery in health anxiety. For example, Mindfulness techniques (Segal, Williams, & Teasdale, 2002) may help patients to relate differently to their images, seeing them as images rather than reality, and may also facilitate patients finding alternative response strategies to the maladaptive responses reported in the current sample.

Whilst direct imagery interventions which seek to disconfirm the content or meaning of images may be challenged, the results of this study highlight a number of other areas which could be targeted through direct intervention techniques. First, imagery re-scripting could be used to produce an alternative meaning or outcome. For example, as in suicide where a ‘flash-forward’ of taking an overdose is re-scripted to the alternative outcome of disposing of the tablets (Holmes, Crane, Fennell, & Williams, 2007), the distressing image of being told you are dying could be re-scripted to one of being told that tests revealed no serious illness. Second, an imagery intervention could attempt to change the time scale of the image, so the meaning being challenged is not whether the patient may become ill or die, but the likely time scale of this being in the distant future. For example, the image of viewing yourself at your current age, lying on your death bed could be re-scripted to one of you lying on your death bed as an elderly person.

Finally, re-scripting could attempt to identify and update the distressing meaning attached to the image and any memories associated with it, as has been demonstrated to be effective in patients with social phobia (Wild et al., 2007, 2008). For example, Wells and Hackmann (1993), identified the meaning of their health anxious patients’ images to reflect negative core beliefs such as ‘I’m bad/inadequate/useless’ and were able to link these meanings to memories of negative early experiences such as being abused or criticised. Wild et al. (2007, 2008) recommend using standard cognitive restructuring techniques to arrive at a new perspective on the event in the memory and linking this new perspective with the memory using imagery re-scripting techniques (e.g., having your adult self enter the image and add the new perspective i.e., interpreting the abuse or criticism as reflecting negatively on the perpetrator rather than the victim, and attempting to put the situation right for the child).

### Limitations and conclusion

This exploratory study is limited by its retrospective nature and it is possible that the social context and demand characteristics of the interview may have affected participants’ responses. Hence replication and extension (e.g., to include appropriate comparison groups) is required to corroborate findings and confirm specificity to health anxiety. Given the episodic nature of health anxiety, future research may also usefully employ prospective designs to examine changes in the content or frequency of images over time.

Given these limitations, the results show that intrusive imagery is a common and distressing phenomenon in health anxiety and may link to the development and maintenance of the disorder. It is hoped that future research into this previously neglected area may provide further insight into the maintenance of the disorder and be used to enhance the efficacy of treatment protocols for patients with health anxiety.

## Figures and Tables

**Table 1:** Demographics and measures of health anxiety, depression and anxiety for participants who did and did not report experiencing intrusive imagery, and for the group as a whole.

	Intrusive images (*n* = 43) Mean (SD)	No intrusive images (*n* = 12) Mean (SD)	All participants (*n* = 55) Mean (SD)
Female %	74.4	83.3	76.36
Age in years	41.86 (12.16)	43.83 (12.01)	42.29 (12.04)
Caucasian %	97.7	100	98.29
Marital status %			
Married	60.5	50	58.2
Single	27.9	25	27.3
Separated or divorced	9.4	0	7.2
Living with a partner	2.3	25	7.3
Education in years	15.49 (4.09)	14.17 (5.36)	15.2 (4.37)
Employment status (%)			
Full/part time employment	67.4	66.6	67.2
Self employed	4.7	0	3.7
Sick leave	9.3	0	7.3
Unemployed	9.3	16.7	10.9
Retired	9.3	16.7	10.9
Duration of current episode (in years)	9.65 (9.83)	8.10 (10.29)	9.32 (9.86)
WI^a^	50.34 (10.80)	47.42 (13.47)	49.7 (11.37)
SHAI^b^	34.53 (7.73)	32.92 (12.16)	34.18 (9.78)
BDI^c^	21.74 (13.85)	19.17 (9.69)	21.18 (13.02)
BAI^d^	19.30 (11.49)	22.75 (11.16)	20.05 (11.4)

a Whitely Index.

b Short Health Anxiety Inventory.

c Beck Depression Inventory.

d Beck Anxiety Inventory.

**Table 2:** The nature of intrusive imagery in patients with health anxiety: Themes and example quotes.

Theme description and number of images in theme	Example images^a^	Example quotes^a^
Impact of own death or serious illness on loved ones (includes 21 images)	Seeing their children and partner “destroyed” due to them having contracted aids.	“My children are crying, my partner is crying- all because I’ve destroyed them with AIDS. Everything is black and it is dark. It is all a mess.”
	Imagining their own funeral and the impact this would have on the children.	“It is my funeral. I imagine how the children would deal with it. My daughter and little boy are there holding hands, my daughter being a surrogate mother.”
	Explaining to her young son that she has a terminal illness and won’t be around for him.	“I imagine how I would tell my little boy and deal with that. I see myself in the house trying to explain that I am not going to be around.”
Suffering from a serious or life-threatening illness (loss of capacity, control and Identity) (includes 20 images)	Imagining self being taken to hospital due to a heart attack and having open heart surgery.	“Having a cardiac arrest. I picture the ambulance coming for me, it takes me to hospital. I see me in hospital – unclogging my arteries, giving me open heart surgery.”
	Imagining being hopeless, desperate and out of control due to having AIDS.	“What it would be like to have AIDS. Not being able to get drug treatment. Feeling hopeless and desperate. Not able to move or escape the pain. I can do nothing. I have no control.”
	Imagining the impact of being barrier nursed in an isolation unit.	“I’m in hospital, in an isolation unit being barrier nursed. I feel claustrophobic, trapped and helpless. I am unable to communicate, fearful and cut off from people. I am unable to live a normal live.”
Death and dying due to physical illness (includes 13 images)	Picturing self dying in a hospice.	“I picture death – I am about to die. I picture myself morbidly ill in a hospice, in a bed.”
	Imagining self lying on a death bed.	“I see myself dying on a death bed – my family are distressed, I am afraid of death and the last moment. It is a stereotypical moment of death.”
	Seeing self dying in a hospital bed.	“I’ve actually seen myself dying, you know, *actually* dying….I’m always in a hospital bed, everyone’s always around me, it’s very much something out of a film really, just lots of screaming and crying.”
Being told “the bad news” – that you have a serious or life-threatening illness (includes 4 images)	Visualising being told by a doctor that they have cancer.	“I visualise being told ‘the bad news’ by the doctor – that I have cancer. I see myself trying to deal with the situation”
	Imagining being told that they have a terminal illness and don’t have long to live.	“Imagining being told ‘the bad news’ and imagining how I would go about it. Me being told I’m ill – being devastated. Not any particular illness. I’ve not got long to live.”
	Imagining being in hospital, being told that they have an incurable terminal illness.	“I am being told I have a terminal illness. There is no cure. I am at hospital and the doctor is giving me the bad news.”

a Identifying details have been removed to preserve participant anonymity.
